# Construction, characterization, and application of immature peach powder-stabilized Pickering emulsion gel

**DOI:** 10.3389/fnut.2025.1643308

**Published:** 2025-08-08

**Authors:** Limin Wu, Yalong Liang, Ninghai Lu, Benguo Liu

**Affiliations:** ^1^School of Resource and Environment, Henan Institute of Science and Technology, Xinxiang, China; ^2^School of Food Science, Henan Institute of Science and Technology, Xinxiang, China

**Keywords:** immature peach powder, Pickering emulsion, particle properties, microstructure, butter substitute

## Abstract

This study utilized immature peach powder (IPP) as a stabilizer to construct a food-grade Pickering emulsion gel system. The effects of IPP concentration (w) and oil phase volume fraction (ϕ) on its structure and properties were investigated, with subsequent application as a butter substitute in cake production. Results indicated that IPP exhibited diverse morphologies and broad size distribution (5–96 μm), with a three-phase contact angle of 75.62° ± 1.27°, demonstrating potential for stabilizing Pickering emulsions. The O/W-type Pickering emulsion gels with *ϕ* ≥ 60% were achieved at *w* ≥ 3%. Droplet size was negatively correlated with w and positively correlated with ϕ, while gel strength showed positive correlations with both parameters. All emulsion gels displayed solid-like viscoelastic behavior and pseudoplasticity in rheological tests. The gel prepared at *w* = 4% and *ϕ* = 60% successfully partially replaced butter in cakes while maintaining normal texture and structure. These findings can promote the application of IPP in foods.

## 1 Introduction

Peaches are extensively cultivated and highly prized fruits. They are abundant in dietary fiber, vitamins, potassium, and polyphenols, and exhibit nutritional functions including antioxidation, blood sugar regulation, and enhancement of intestinal health (1). In the course of peach growth and development, without any management measures, the natural fruit drop rate can range from 60%−80% (2). According to data from the United States Department of Agriculture (USDA), the average fruit drop rate in California orchards, a major global peach-producing region, and ranges from 40%−60%. Consequently, growers annually perform manual fruit thinning operations. This practice not only directs nutrients to retained fruits for enhanced individual enlargement and improved commercial value, but also prevents excessive fruiting that could lead to insufficient flower bud differentiation in the following year, thereby stabilizing yields. The fruits obtained from commercial thinning operations conducted ~45 days after full bloom are immature peaches. Research reveals that immature peaches contain abundant nutrients and functional components including polyphenols, carbohydrates, and proteins, exhibiting potential applications in preventing and managing cardiovascular diseases, type II diabetes, enteritis, tumors, and neurological disorders (3–5). Immature peaches contain significantly higher polyphenols (up to 48.7 mg GAE/g DW) and antioxidant capacity than ripe fruit (6), making them ideal for functional food development. Currently, as an agricultural by-product of peach cultivation, immature peaches lack industrial-scale utilization, and are primarily disposed through natural degradation or as waste. Therefore, developing high-value products from immature peaches could not only facilitate effective recycling of these premature fruits but also advance sustainable development within the peach industry.

Pickering emulsions are colloidal systems stabilized by solid particles that irreversibly adsorb at the oil-water interface through micro- or nano-scale particles, forming robust spatial barriers to prevent droplet aggregation and coalescence. Recognized for their high stability, low toxicity, and biocompatibility, these emulsions find extensive applications in pharmaceuticals, cosmetics, and food industries (7). Although numerous studies have reported plant-derived extracts (proteins, polysaccharides, etc.) as emulsifiers for Pickering stabilization, their extraction and purification processes remain time-consuming, labor-intensive, and environmentally unfriendly (8, 9). Direct utilization of raw plant particles as Pickering emulsifiers shows significant potential for cost reduction and enhanced product naturalness. Hollestelle et al. demonstrated that unrefined apple pomace powder could stabilize Pickering emulsions with large droplet diameters (up to 80 μm) and low surface coverage, yet maintaining coalescence-free stability during storage. This stabilization mechanism involves soluble components governing particle dimensions while insoluble fractions facilitate oil entrapment through 3D network adsorption (10). Similarly, Liao et al. (11) developed soybean meal-based Pickering emulsions, revealing that moderate increases in particle concentration promoted uniform small droplet formation, with compact interfacial adsorption layers preventing droplet aggregation, thereby achieving high elasticity and improved printability. Zhou et al. (12) utilized tea residue powder to stabilize Pickering emulsions, demonstrating that high-pressure homogenization treatment could enhance their emulsification stability. Immature peach is the immature fruit of the peach tree, rich in dietary fiber, pectin, proteins, and polyphenols, exhibit inherent Pickering emulsification potential. However, systematic investigations on the construction, characterization, and application of immature peach powder (IPP)-stabilized Pickering emulsion gels remain unexplored (13–15). To bridge this knowledge gap, we developed a food-grade Pickering emulsion gel system utilizing immature peach powder (IPP) as particulate stabilizers, and systematically investigated the synergistic effects of IPP concentration (w) and oil phase fraction (ϕ) on gel properties, with subsequent validation of its functionality as a butter substitute in cakes.

## 2 Materials and methods

### 2.1 Materials and chemicals

Immature peaches (*Prunus persica* var. *nucipersica* cv. “Zhongyou No. 4”) were collected during scheduled fruit thinning at 45 ± 5 days after full bloom (DAFB) from an orchard in Qixian County. Only fruits meeting the above morphological criteria were processed. They were freeze-dried, ground, and passed through a 200-mesh sieve to obtain immature peach powder (IPP). IPP was sealed in polyethylene zip bags and stored in desiccators with silica gel maintaining 15 ± 5% relative humidity at 25 ± 2°C. All samples were processed within 48 h to prevent bioactive compound degradation. Medium-chain triglycerides (MCT) were obtained from Yuanye Bio-Technology Co., Ltd (Shanghai, China). Cake flour was provided by Liangrun Whole Grain Foods Co., Ltd (Xinxiang, China). The butter used was Anchor brand butter from Fonterra Cooperative Group (Auckland, New Zealand).

### 2.2 Particle size determination of IPP

The particle size characteristics of IPP in aqueous phase were characterized by laser diffraction method. Measurements were performed using a Malvern Mastersizer 2000 laser particle size analyzer (Malvern Panalytical Ltd., UK), which operates based on static light scattering principle. An appropriate amount of IPP was weighed into the measurement cell, dispersed in deionized water, and subjected to ultrasonication (40 kHz, 300 W) to ensure homogeneous dispersion. The optical parameters were set according to Mie scattering theory: sample refractive index 1.760 with absorption rate 0.1; dispersant (deionized water) refractive index 1.330. During measurement, the obscuration was maintained within the optimized range of 15%−16%. After data acquisition, the results were processed through the Universal Analysis mode for deconvolution, yielding volume-weighted particle size distribution curves and characteristic particle size parameters including D_10_, D_50_, and D_90_ (16).

### 2.3 Contact angle measurement of IPP

Three-phase contact angles were quantified using an optical tensiometer (Attension Theta Lite, Biolin Scientific AB, Sweden) with automated liquid dispensing capability. IPP was compressed into standardized test discs and placed in a clean quartz Petri dish (50 × 50 mm). A defined volume of MCT was injected to create a liquid-sealed environment. Using an automated dosing module (accuracy: ± 0.1 μL), a 6 μL ultrapure water droplet was precisely dispensed. A high-speed camera was simultaneously activated to record the evolution of the three-phase contact interface from 0 to 12 s. The time-dependent contact angle variation curve was calculated using One Attension v5.0 software based on the Young-Laplace equation curve-fitting algorithm. The average value from three parallel measurements during the stable phase (8–12 s) was adopted as the final characteristic value (17).

### 2.4 Microscopic observation of IPP

The morphological analysis of IPP was conducted using a ZEISS Sigma 300 field emission environmental scanning electron microscope (Oberkochen, Germany). The sample was uniformly dispersed and mounted on an aluminum stub, followed by platinum sputter coating using an ion sputter coater (EMS150T, Leica, Germany) to enhance conductivity. The analysis was performed under low vacuum mode at an accelerating voltage of 5.0 kV with 500 × magnification, where secondary electron images were acquired. This approach effectively minimized the influence of surface charge accumulation on imaging quality (18).

### 2.5 Fabrication of Pickering emulsion gel

To investigate the synergistic regulatory mechanism of IPP concentration (w, 1.0%−4.0%) and oil phase volume fraction (ϕ, 40%−70%) on emulsion gel formation capability, a multi-factor experimental system was designed using phase composition analysis. Predetermined proportions of medium-chain triglycerides (MCT) were mixed with deionized water containing specific amounts of IPP. The mixtures were then homogenized using an IKA Ultra-Turrax T18 disperser (Staufen, Germany) at room temperature with shear homogenization (12,000 rpm, 3 min) to construct Pickering emulsion gels (19).

### 2.6 Droplet size measurement of Pickering emulsion gel

Following the methodology described in Section 2.5, representative Pickering emulsion gels stabilized by IPP were prepared (*w* = 2.0%−4.0%, ϕ = 70%; *w* = 4.0%, ϕ = 50–70%). The droplet size distribution was determined using a BT-9300H laser particle size analyzer (Dandong, China). Prior to measurement, the emulsions were appropriately diluted with deionized water as the dispersion medium. The analysis was conducted when the system's obscuration stabilized within the 15%−16% range, with the initial obscuration strictly maintained below 2% to ensure measurement accuracy (20).

### 2.7 Gel strength measurement of Pickering emulsion gel

The gel strength was measured using a TA-XT plus texture analyzer (Stable Micro Systems Ltd., UK) equipped with a P0.5 cylindrical probe, following the GMIA method. The test parameters were set as follows: probe speed of 1.0 mm/s, trigger threshold of 3.0 g, and compression displacement of 4 mm. Data were processed using Exponent 6.1 software (21).

### 2.8 Rheological characterization of Pickering emulsion gel

The dynamic rheological properties of emulsion gels were investigated using a HAAKE MARS III rotational rheometer (Thermo Scientific, Germany) equipped with P35TiL parallel-plate geometry. With a fixed gap of 1 mm, samples were equilibrated at 25°C for 60 s prior to measurements. The amplitude sweep test (*f* = 1 Hz, τ = 1–100 Pa), frequency sweep (τ = 5 Pa, *f* = 0.1–100 Hz), and shear rate sweep test (γ = 0.1–20 s−^1^) were sequentially performed. All experimental data were acquired and processed using RheoWin software (22, 23).

### 2.9 Cake preparation method

IPP-stabilized Pickering emulsion gel (*w* = 4%, *ϕ* = 60%) was used to investigate the effect of butter replacement levels on cake formation and quality. As shown in Table 1, ingredients were precisely weighed according to butter substitution ratios (RBS = 0, 20, 40, 60, 80, and 100%) and thoroughly mixed. The batter was homogenized using an LD-133 electric mixer. The homogeneous batter was then transferred into standard baking molds (15 cm × 8 cm × 7 cm) and baked in a ZCV30 steam convection oven (170°C for 40 min). After baking, the cakes were equilibrated at room temperature for 2 h before subsequent analysis. The cakes prepared at RBS = 0, 20, 40, 60, 80, and 100% were designated as C-R0, C-R20, C-R40, C-R60, C-R80, and C-R100, respectively.

**Table 1:** Cake formulations.

**Cake**	**C-R0**	**C-R20**	**C-R40**	**C-R60**	**C-R80**	**C-R100**
**RBS (%)**	**0**	**20**	**40**	**60**	**80**	**100**
Pastry flour (g)	100	100	100	100	100	100
Butter (g)	100	80	60	40	20	0
Emulsion gel (g)	0	20	40	60	80	100
Sugar (g)	100	100	100	100	100	100
Whole egg liquid (g)	100	100	100	100	100	100
Baking powder (g)	2	2	2	2	2	2

### 2.10 Cake appearance evaluation

The cake volume was determined using the millet displacement method, and the weight was measured to calculate density and specific volume. Color parameters (L^*^, a^*^, b^*^) were measured using a Konica Minolta CR-400 colorimeter.

### 2.11 Cake texture and structure evaluation

The cake samples were cut into 20 mm × 20 mm × 20 mm cubes for texture profile analysis (TPA) using a TA-XT Plus texture analyzer equipped with a P/36R probe. Additionally, 20 mm-thick standardized cross-sections were prepared and analyzed for internal structure using a C-Cell digital imaging system (Calibre Control International Ltd., Warrington, UK) (24).

### 2.12 Statistical analysis

All measurements were performed in triplicate. Data were analyzed for significant differences using IBM SPSS Statistics 26 software (IBM Corp., USA) and expressed as mean ± SD. Duncan's multiple test was employed for *post-hoc* comparisons (*p* < 0.05). Graphical representations were generated using Origin 2018 software (OriginLab Corp., USA).

## 3 Results and discussion

### 3.1 Particle size of IPP

**Figure 1:**
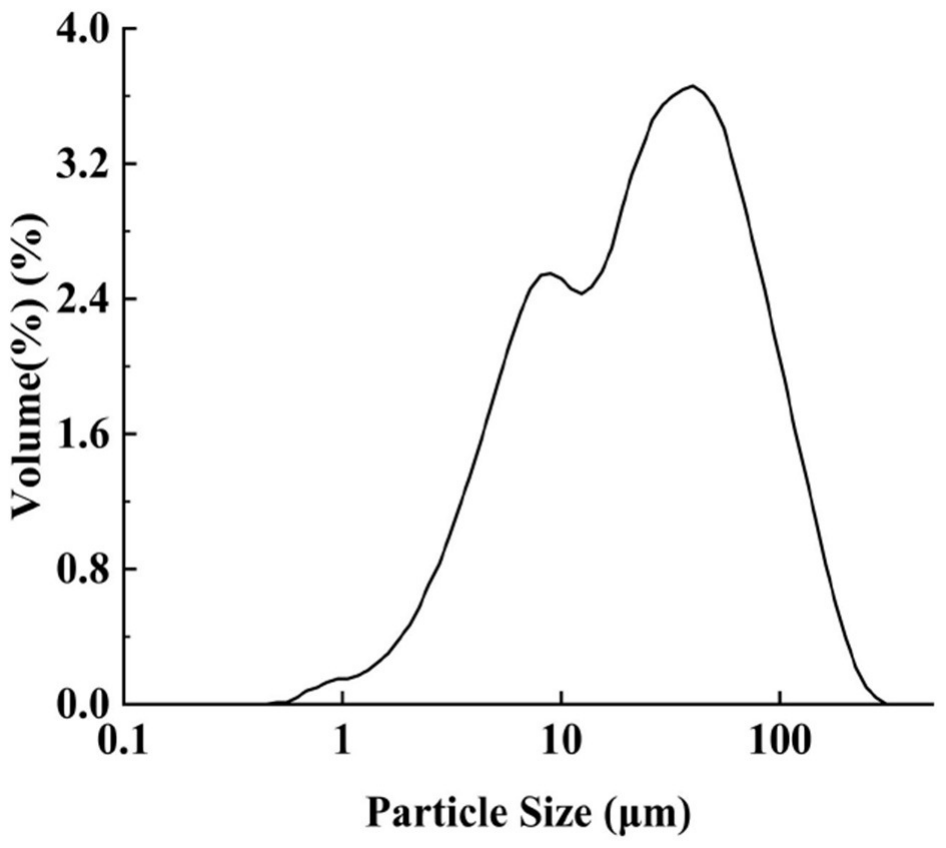
Size distribution of IPP.

The size of Pickering particles is a critical factor affecting emulsion stability. Larger particles may fail to adsorb effectively at the oil-water interface, leading to reduced emulsion stability, whereas smaller particles generally stabilize emulsions more efficiently by forming a denser interfacial layer (25, 26). However, excessively small particles may increase Brownian motion, promoting aggregation and thereby compromising stability (27). The measurement results (Figure 1) indicate that IPP exhibit a broad size distribution, with d_0.1_ = 4.842 μm, d_0.5_ = 26.3 μm, and d_0.9_ = 96.22 μm, suggesting relatively large particle sizes and significant polydispersity. This characteristic has dual implications for the stability of oil-in-water (O/W) emulsions. On one hand, larger particles (>10 μm) may lead to emulsion stratification or sedimentation due to insufficient interfacial adsorption and gravitational effects, particularly when the particles cannot form a compact barrier at the oil-water interface (28). On the other hand, moderate particle polydispersity might enhance emulsion stability through steric hindrance, provided the particles possess appropriate wettability (hydrophilic-lipophilic balance) for stable interfacial adsorption (29). The stabilization mechanisms of Pickering emulsions demonstrate a non-linear dependence on particle size distribution. Notably, particle sizes exceeding optimal thresholds (quantified by d_0.9_ values in our experimental system) significantly compromise interfacial adsorption kinetics, predisposing the system to gravitational destabilization through phase segregation. This mechanistic relationship stands in marked contrast to the performance characteristics of submicron particulates, which exhibit superior efficacy in stabilizing reduced-diameter emulsion droplets through enhanced interfacial packing density, ultimately ensuring colloidal stability (30).

### 3.2 Wettability of IPP

**Figure 2:**
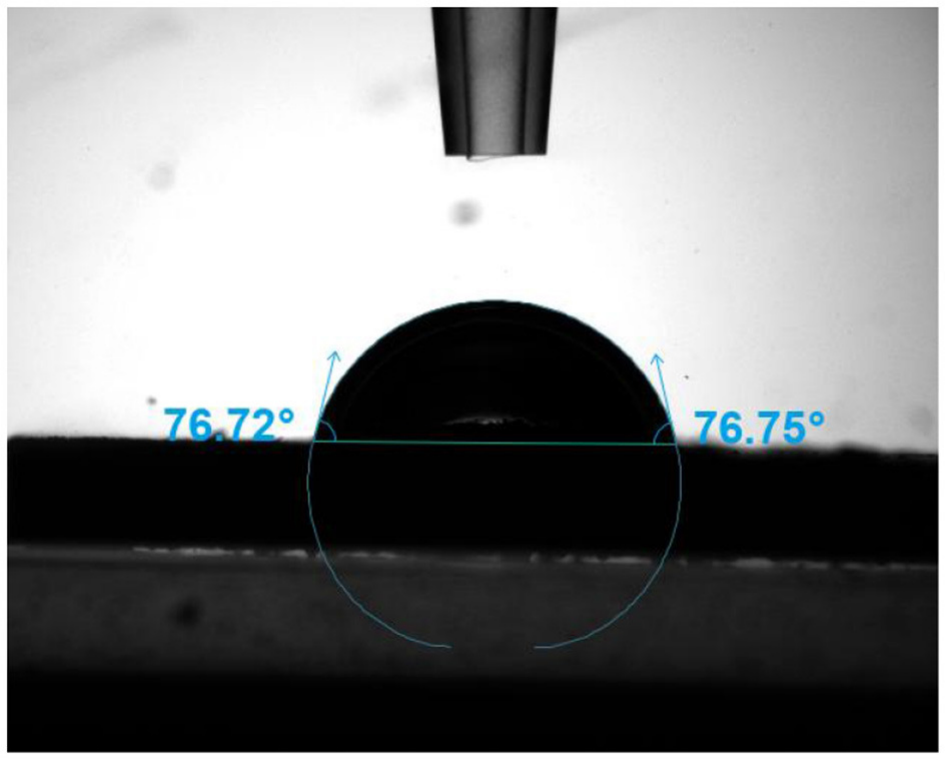
Contact angle of IPP.

Solid particles are crucial for the formation and stability of Pickering emulsions, and their wettability determines their positioning at the oil-water interface, thereby influencing emulsion stability and type. Wettability is typically characterized by the three-phase contact angle (θ). When θ < 90°, the particles are mainly located in the aqueous phase, resulting in an O/W emulsion; when θ > 90° the particles are predominantly in the oil phase, leading to a W/O emulsion; when θ = 90°, the particles can accumulate in both phases, thereby forming W/O and O/W emulsions with higher stability (31). When θ approaches 90°, the interfacial adsorption energy is maximized, forming a stable interfacial layer and significantly enhancing the emulsion's resistance to coalescence (25, 32). In Figure 2, the three-phase contact angle of IPP particles was 75.62° ± 1.27°, indicating moderate amphiphilicity. This wettability was essential for the formation and stability of oil-in-water (O/W) emulsions. A contact angle of 75.62° suggested that the particle surface was slightly more hydrophilic, which may lead to insufficient interfacial adsorption stability. Therefore, additional stabilization mechanisms, such as interparticle interactions or continuous-phase viscosity, might be necessary. For instance, Li et al. (33) found that micron-sized particles (>10 μm) with moderate wettability could stabilize emulsions through dual mechanisms: steric hindrance and interfacial reinforcement. However, gravitational sedimentation-induced phase separation must be avoided. Combined with particle size analysis (d_0.5_ = 26.3 μm), the relatively large particle size may reduce interfacial coverage. Nevertheless, the moderate wettability could partially compensate for this drawback by promoting particle aggregation into a three-dimensional network structure, thereby delaying phase separation.

### 3.3 Appearance of IPP

**Figure 3:**
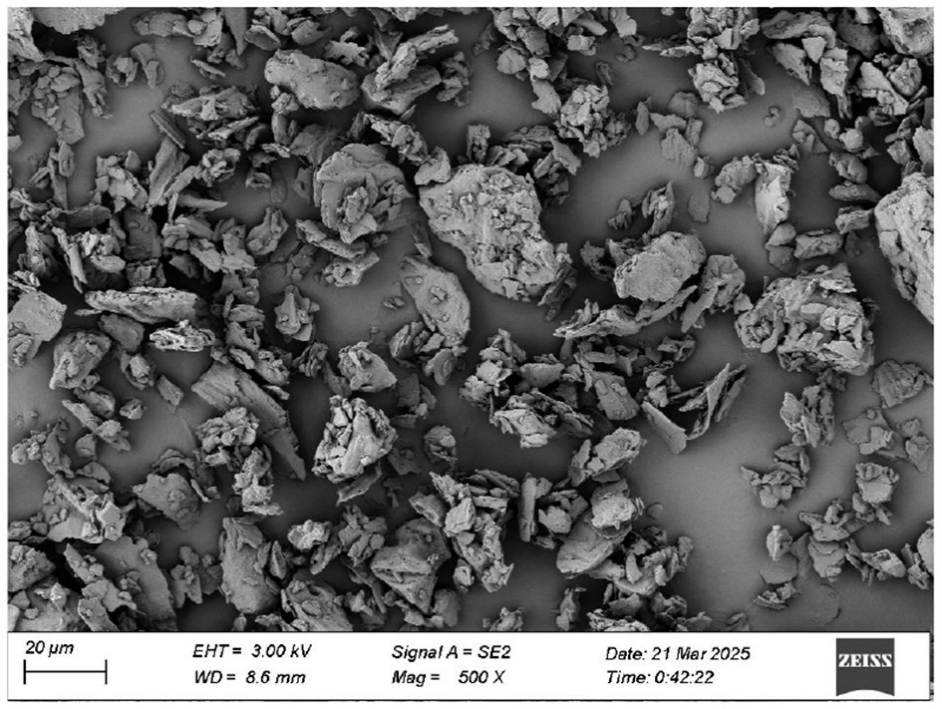
Scanning electron micrograph of IPP.

The morphological features of particles serve as a critical determinant for the interfacial stability of Pickering emulsions. Their geometric configuration and polymorphic differences directly influence the molecular packing density at the interface, thereby governing the rheological properties, phase separation behavior, and macroscopic stability of the emulsion system. While conventional research has predominantly focused on symmetric spherical particles, recent advances highlight the unique advantages of anisotropic particles (e.g., ellipsoids, polyhedrons, rod-like crystals, fibrous assemblies, and nanogel aggregates) in interfacial engineering (34). It was reported that non-spherical particles tended to orient at the oil/water interface with their long axes parallel to the interface, suggesting stronger adsorption (35). As shown in Figure 3, IPP, being a mixture, exhibited an irregular block-like structure with micron-scale surface roughness. When solid particles acted as interfacial stabilizers, surface roughness enhanced their anchoring capability at the oil-water interface, forming a mechanical barrier that suppresses droplet coalescence. It is hypothesized that such structural characteristics may improve emulsion stability via the Pickering mechanism. Additionally, a uniform particle size distribution facilitates the formation of a densely packed interfacial layer, reducing interfacial tension, and thereby enhancing kinetic stability (36). Literature suggests that the hydrophobicity of plant-derived particles correlates with their surface porosity, and the porous nature of IPP may enhance lipid adsorption capacity, further improving dispersion efficiency during emulsion homogenization (37). In conclusion, the morphological traits of IPP provided a physical foundation for its role as a natural emulsifier, though further emulsification experiments were required for validation.

### 3.4 Formation of IPP-stabilized Pickering emulsion gels

**Figure 4:**
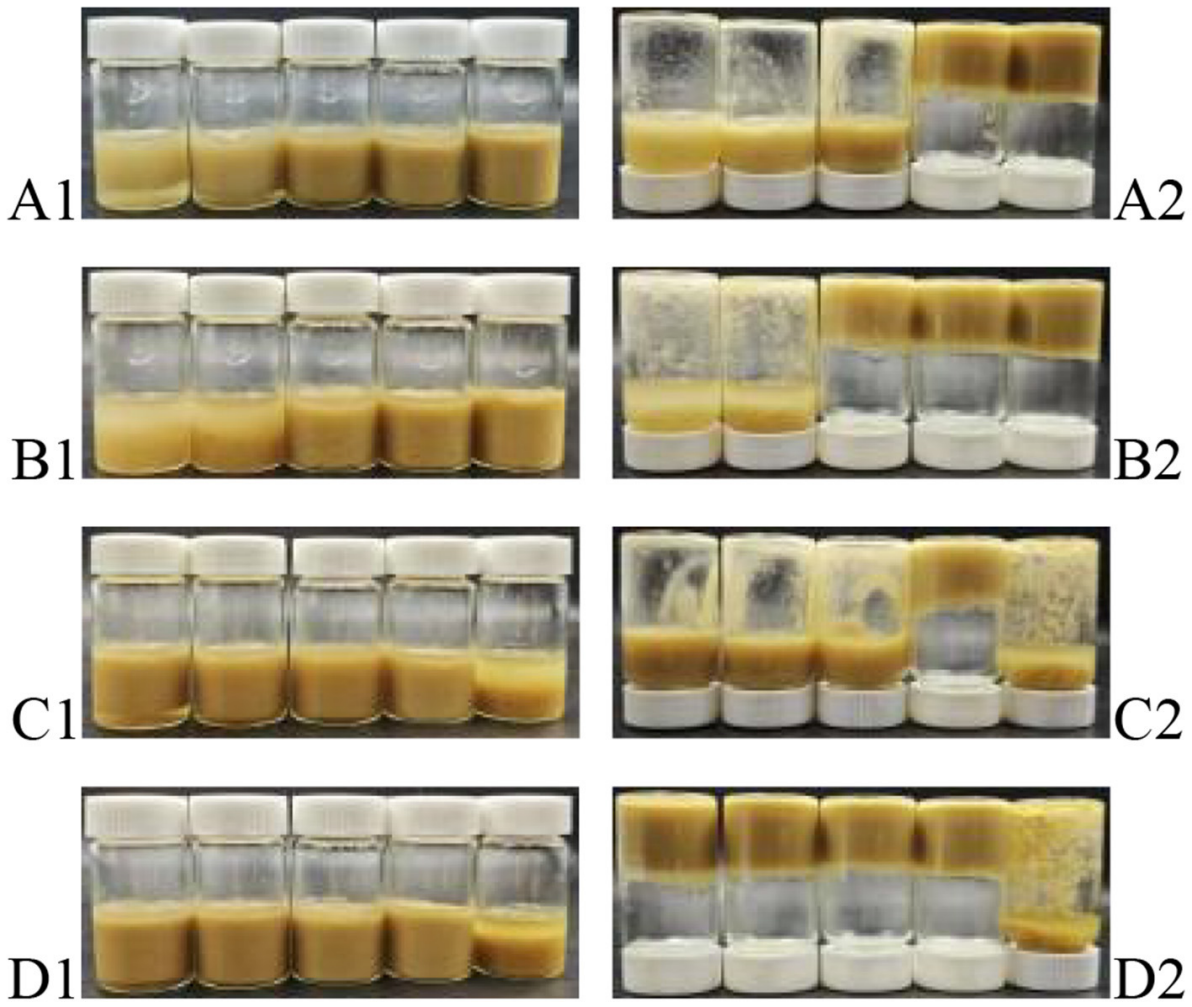
Pickering emulsions stabilized by IPP **(A1)** ϕ = 50%, *w* = 0.5%, 1%, 2%, 3%, 4%; **(B1)** ϕ = 70%, *w* = 0.5%, 1%, 2%, 3%, 4%; **(C1)** *w* = 2.0%, ϕ = 40%, 50%, 60%, 70%, 80%; **(D1)** *w* = 4.0%, ϕ = 40%, 50%, 60%, 70%, 80%; **(A2–D2)** show the inverted states after 2 h for samples, **(A1–D1)** that had been stored for 24 h, respectively.

The influence of oil phase volume fraction (ϕ) and IPP concentration (w) on emulsion gel formation was systematically analyzed using vial inversion tests to assess the feasibility of IPP-stabilized Pickering emulsion gels (Figure 4). At a low oil phase (ϕ = 50%), increasing w from 0.5%−4% transformed the system from a flowable emulsion (*w* = 0.5%) to a solid-like gel (*w* = 4%). After 24-h storage, samples with higher w (≥2%) exhibited no flow upon inversion (Figure 4A2), indicating that IPP effectively suppressed droplet coalescence through interfacial adsorption and three-dimensional network formation. Under high oil phase conditions (ϕ = 70%), gel rigidity significantly increased at equivalent w values (Figure 4B1 vs. Figure 4A1). However, at *w* = 0.5%, insufficient particle coverage led to pronounced phase separation (Figure 4B2), confirming that higher oil fractions demand greater particle concentrations for interfacial stability (38). Further investigation revealed that at fixed *w* = 2%, ϕ = 80% gels suffered from network fracture due to overcrowded oil droplets (Figure 4C1), resulting in structural collapse upon inversion (Figure 4C2). In contrast, the systems with *w* = 4% (Figure 4D1) maintained long-term stability even at ϕ = 80% through redundant particle network encapsulation (Figure 4D2), aligning with recent “particle-to-oil volume ratio threshold” theories (39). It could be concluded that IPP synergistically enhanced the mechanical strength and deformation resistance of high-oil emulsion gels through dual interfacial stabilization and network reinforcement mechanisms. These findings provide a theoretical foundation for formulating high-oil-phase emulsion gels, demonstrating particular promise for low-fat food development and functional ingredient encapsulation systems.

### 3.5 Droplet size analysis of Pickering emulsion gels

Droplet size serves as a critical microstructural parameter of emulsion systems, directly influencing their functional properties and stability. Studies demonstrate that particle type, concentration, and oil phase volume fraction constitute core factors regulating droplet behavior. It was found that reducing droplet size significantly enhanced the mechanical strength and water-holding capacity of emulsion gels (40). Similarly, Xiao et al. (41) achieved synergistic optimization of droplet size and rheological properties by modulating chitosan-caseinate composite particle concentrations. When fabricating high internal phase emulsions using zein nanoparticles, Cui et al. (42) observed that increasing particle concentration not only reduced droplet diameter but also markedly improved gel hardness. As presented in Table 2, at a fixed oil phase volume fraction (ϕ = 70%), the particle size decreased with increasing peach powder content (w, 2%−4%). Conversely, at a fixed peach powder content (*w* = 4%), the particle size increased with increasing oil phase volume fraction (ϕ, 50%−70%). This aligns with Song et al. who reported that octenyl succinic anhydride-modified soy protein-stabilized emulsions exhibited enlarged droplets at higher oil ratios. However, demonstrated an inverse trend in tea polyphenol-whey protein composite particle systems, where increased oil phase induced droplet refinement attributable to particles' differential affinity for continuous vs. dispersed phases (43). While higher particle concentrations universally promote droplet size reduction, the impact of oil phase volume fraction depends critically on particles' surface chemistry and interfacial partitioning behavior. These findings provide mechanistic insights for tailoring emulsion microstructure through particle engineering.

**Table 2:** Emulsion droplet diameter distribution.

**ϕ (%)**	***w* (%)**	**D_10_ (μm)**	**D_50_ (μm)**	**D_90_ (μm)**
70%	2	31.32 ± 1.00^g^	90.42 ± 0.85^d^	177.17 ± 2.80^a^
	3	27.25 ± 0.52^h^	84.77 ± 1.02^e^	150.47 ± 3.85^b^
	4	22.90 ± 0.04^i^	74.98 ± 1.90^f^	142.20 ± 1.31^c^
***w*** **(%)**	ϕ **(%)**	**D**_10_ **(**μ**m)**	**D**_50_ **(**μ**m)**	**D**_90_ **(**μ**m)**
4%	50	6.96 ± 0.67^i^	44.49 ± 1.64^f^	92.18 ± 3.79^d^
	60	14.77 ± 2.45^h^	64.36 ± 0.30^e^	123.97 ± 0.71^b^
	70	22.90 ± 0.04^g^	74.98 ± 1.90^d^	142.20 ± 1.31^a^

Different superscript lowercase letters indicate statistically significant differences among groups (p < 0.05).

### 3.6 Gel strength of Pickering emulsion gels

**Figure 5:**
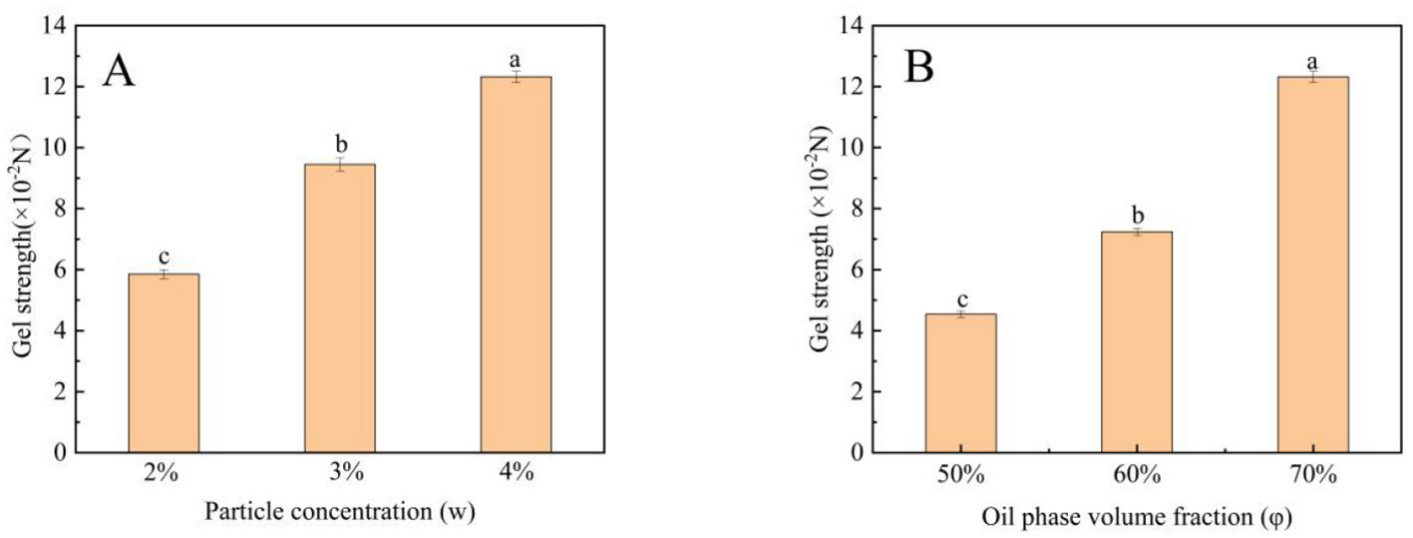
Effect of particle concentration **(A)** and Oil phase volume fraction **(B)** on gel strength of Pickering emulsion gels. Bars with different letters differ significantly at *p* < 0.05.

Emulsion gels, as a novel soft matter material, have demonstrated significant potential in applications such as fat replacement, nutrient encapsulation, and texture regulation. In recent years, plant protein- or polysaccharide-based emulsion gels have garnered considerable attention due to their environmental sustainability and tunable functionalities. However, their mechanical properties are notably influenced by oil phase ratios and particle packing behaviors. Studies indicate that high ϕ enhance network compactness through interfacial adsorption, while the incorporation of active particles can act as physical crosslinking points. However, excessive particle addition may induce aggregation. Therefore, the synergistic interaction between oil and particles and its dynamic impact on gel strength require further investigation. As shown in the Figure 3, w and ϕ exhibited a clear positive correlation with gel strength. When w increased from 2% to 4%, gel strength rose linearly (Figure 5A), aligning with the particle-dominated “percolated network” theory. As ϕ increased from 50% to 70%, gel strength improved from 4.54 × 10^−2^ to 12.32 × 10^−2^ N (Figure 5B), attributed to the expansion of oil droplet interfacial active areas and increased particle adsorption density. This trend was consistent with prior findings in Tartary buckwheat bran-stabilized emulsion gels, where particle addition similarly enhanced gel strength (44).

### 3.7 Rheological properties of Pickering emulsion gels

**Figure 6:**
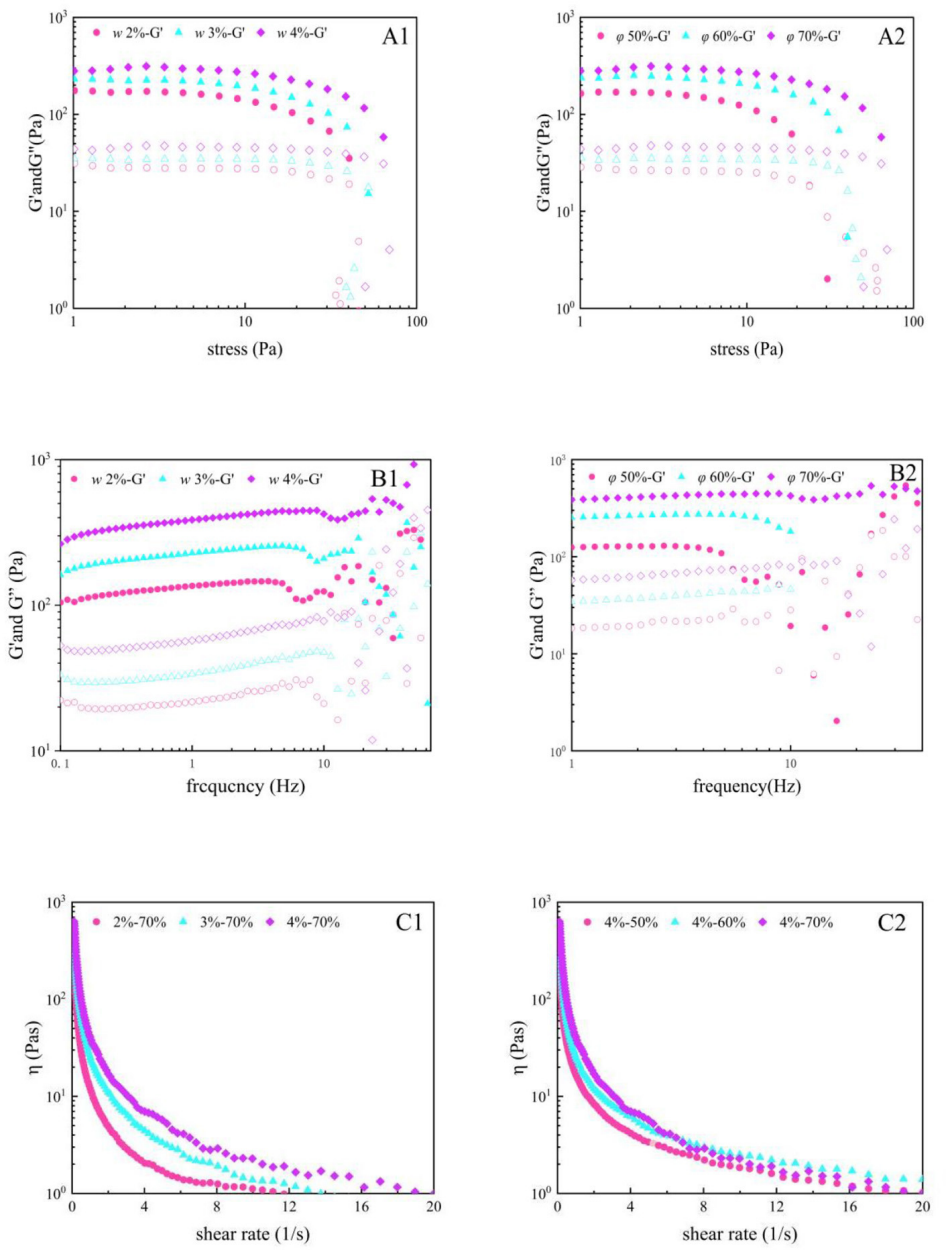
Effect of w and ϕ on the stress sweep **(A)**, frequency sweep **(B)**, and apparent viscosity **(C)** of Pickering emulsion gels (a, b, and c were prepared at ϕ = 70%, *w* = 2%, 3%, and 4%, respectively; d, e, and f were prepared at *w* = 4%, ϕ = 50%, 60%, and 70%, respectively).

The rheological properties of Pickering emulsion gels are critical for their applications in food processing, and their behavior can be systematically characterized through oscillatory stress sweeps, frequency sweeps, and shear rate sweeps. Studies demonstrate that solid particles in the emulsion form a rigid adsorption layer at the oil-water interface, promoting tight droplet packing and the construction of a three-dimensional network structure, endowing the system with elastic-dominated solid-like characteristics (G' > G”) (45). As shown in Figures 6A1, A2, within the critical stress range, the storage modulus (G') and loss modulus (G”) remained stable, indicating an intact gel network. However, when the stress exceeded the threshold, the moduli sharply declined, reflecting irreversible collapse of the network structure. Increased w significantly enhanced the elastic modulus (G') of the gel, attributed to strengthened interparticle interactions and densification of the three-dimensional network. Nevertheless, excessive particles may induce localized aggregation, exacerbating shear-thinning effects. Variations in ϕ exhibited nonlinear impacts: moderate increases (e.g., *ϕ* = 60%) optimized oil droplet dispersion and enhance gel stability, whereas excessive ϕ (e.g., 70%) disrupted structural continuity due to oil phase overload. Frequency sweep analysis (Figures 6B1, B2) further confirm that gels with *ϕ* = 60% display optimal solid-like behavior, emphasizing the balance between w and ϕ for network stability. Additionally, shear-thinning behavior in apparent viscosity (Figures 6C1, C2) revealed the sensitivity of the particle network to shear forces. Systems with higher w exhibit greater initial viscosity but faster structural relaxation under shear (46).

### 3.8 Appearance of cake

Cake, as a multiphase food system, can be regarded as an oil-in-water (O/W) emulsion in its unbaked batter state, primarily composed of fat, an aqueous phase, and emulsifiers. When replacing butter with IPP emulsion gel formulated at *w* = 4% and *ϕ* = 60%, the resulting cake constitutes a “plant-based multiphase gel network system,” co-constructed by the emulsion gel phase and the starch-protein matrix (47). As shown in Table 3, substituting butter with IPP emulsion gel significantly impacts the appearance of cake. With the increase in RBS, the volume and specific volume of the cakes gradually decreased, which is attributed to the disruption of the protein network in the dough caused by the emulsion gel. The L^*^ value (Luminance) also progressively decreased with higher substitution rates, likely due to thermal oxidation of polyphenols in IPP and enhanced Maillard reactions caused by restricted water migration within the gel network. The a^*^ value (red-green chromaticity) shifted from a greenish hue toward neutrality, potentially linked to emulsion-mediated inhibition of chlorophyll degradation or accelerated carotenoid oxidation. The b^*^ value (Yellow-blue chromaticity) declined, reflecting the dilution of butter's natural yellow pigments. Notably, color differences remained mild at low substitution rates (RBS ≤ 40%), while significant color deterioration occurs at high substitution rates (RBS > 60%). This may arise from densified gel networks restricting uniform pigment distribution and intensified interfacial reactions. The observed color alterations not only reflect the physicochemical mechanisms of ingredient substitution but also underscore the challenges in balancing sensory appeal and functional performance during the development of low-fat, high-fiber food products.

**Table 3:** Appearance analysis of cakes.

**Parameter**		**C-R0**	**C-R20**	**C-R40**	**C-R60**	**C-R80**	**C-R100**
Loaf volume (cm3/g)		661.20 ± 0.62^a^	635.77 ± 0.45^b^	611.17 ± 0.55^c^	570.93 ± 0.32^d^	544.63 ± 0.61^e^	544.60 ± 0.72^e^
Specific volume (mL/g)		1.89 ± 0.002^a^	1.84 ± 0.002^b^	1.83 ± 0.001^c^	1.74 ± 0.002^d^	1.68 ± 0.002^e^	1.65 ± 0.001^f^
Color	L^*^	75.54 ± 0.26^a^	68.57 ± 0.46^b^	66.49 ± 0.88^c^	64.17 ± 0.26^d^	61.41 ± 0.40^e^	59.70 ± 1.23^f^
	a^*^	−4.64 ± 0.06^f^	−3.00 ± 0.10^e^	−1.81 ± 0.18^d^	−1.08 ± 0.04^c^	−0.67 ± 0.06^b^	−0.20 ± 0.10^a^
	b^*^	38.55 ± 0.80^a^	32.61 ± 0.38^b^	28.76 ± 0.29^c^	27.67 ± 0.26^d^	26.01 ± 0.19^e^	24.23 ± 0.38^f^

Different superscript lowercase letters in the same row indicate statistically significant differences among groups (p < 0.05).

### 3.9 Texture of cake

Emulsion gels, as functional fat substitutes, have garnered significant attention in baked goods in recent years due to their ability to modulate moisture distribution and network structure to improve product texture (48). Textural properties are core indicators for evaluating the quality of baked products, closely linked to sensory experience and process optimization. As shown in Table 4, the substitution of butter with IPP emulsion gel primarily impacted cake texture by increasing hardness, reducing elasticity, causing volume shrinkage, and inducing a nonlinear weight loss trend (initial increase followed by decline). With increasing RBS (0%−80%), the gluten network strengthens due to reduced fat content, while intensified starch gelatinization led to structural densification and a marked rise in hardness. Adhesiveness reached its maximum negative peak at RBS = 40%, indicating uneven internal moisture distribution. This, combined with accelerated moisture evaporation following initial fat network disruption, explains the initial increase in weight loss. At higher substitution rates (RBS ≥ 60%), enhanced cohesiveness and saturated water-holding capacity of the emulsion jointly suppressed moisture migration, resulting in reduced weight loss in later stages. Complete substitution (RBS = 100%) caused a significant decline in elasticity, likely due to the absence of fat's lubricating effects in the emulsion, which restricted gas expansion capacity and ultimately minimized cake volume. Notably, changes in elasticity and cohesiveness were not statistically significant. At substitution rates of WBS ≤ 60%, the texture remained largely stable compared to the control group, demonstrating favorable substitution efficacy.

**Table 4:** Texture analysis of cakes.

**Parameter**	**C-R0**	**C-R20**	**C-R40**	**C-R60**	**C-R80**	**C-R100**
Hardness	1,393.64 ± 220.45^ab^	1,060.49 ± 28.44^b^	1,477.88 ± 149.21^ab^	1,114.98 ± 145.94^ab^	1,545.84 ± 36.44^a^	1,299.6 ± 332.57^ab^
Adhesiveness	−3.38 ± 0.73^ab^	−2.51 ± 0.71^a^	−5.35 ± 0.61^c^	−2.52 ± 1.19^a^	−5.01 ± 0.57^bc^	−1.67 ± 0.20^a^
Springiness	0.82 ± 0.01^a^	0.83 ± 0.01^a^	0.83 ± 0.01^a^	0.84 ± 0.01^a^	0.89 ± 0.03^a^	0.66 ± 0.32^a^
Cohesiveness	0.48 ± 0.03^a^	0.51 ± 0.01^a^	0.52 ± 0.01^a^	0.52 ± 0.02^a^	0.65 ± 0.17^a^	0.65 ± 0.10^a^
Gumminess	673.99 ± 149.98^b^	538.20 ± 2.26^b^	769.37 ± 66.50^ab^	575.87 ± 88.92^b^	997.41 ± 235.27^a^	832.96 ± 85.71^ab^
Chewiness	553.28 ± 117.43^ab^	445.53 ± 2.48^b^	640.18 ± 58.65^ab^	486.47 ± 77.16^ab^	888.74 ± 237.55^a^	561.82 ± 323.35^ab^
Resilience	0.17 ± 0.01^b^	0.18 ± 0.01^b^	0.20 ± 0.01^ab^	0.20 ± 0.01^ab^	0.25 ± 0.07^ab^	0.29 ± 0.08^a^

Different superscript lowercase letters in the same row indicate statistically significant differences among groups (p < 0.05).

### 3.10 Internal structure of cakes

**Figure 7:**
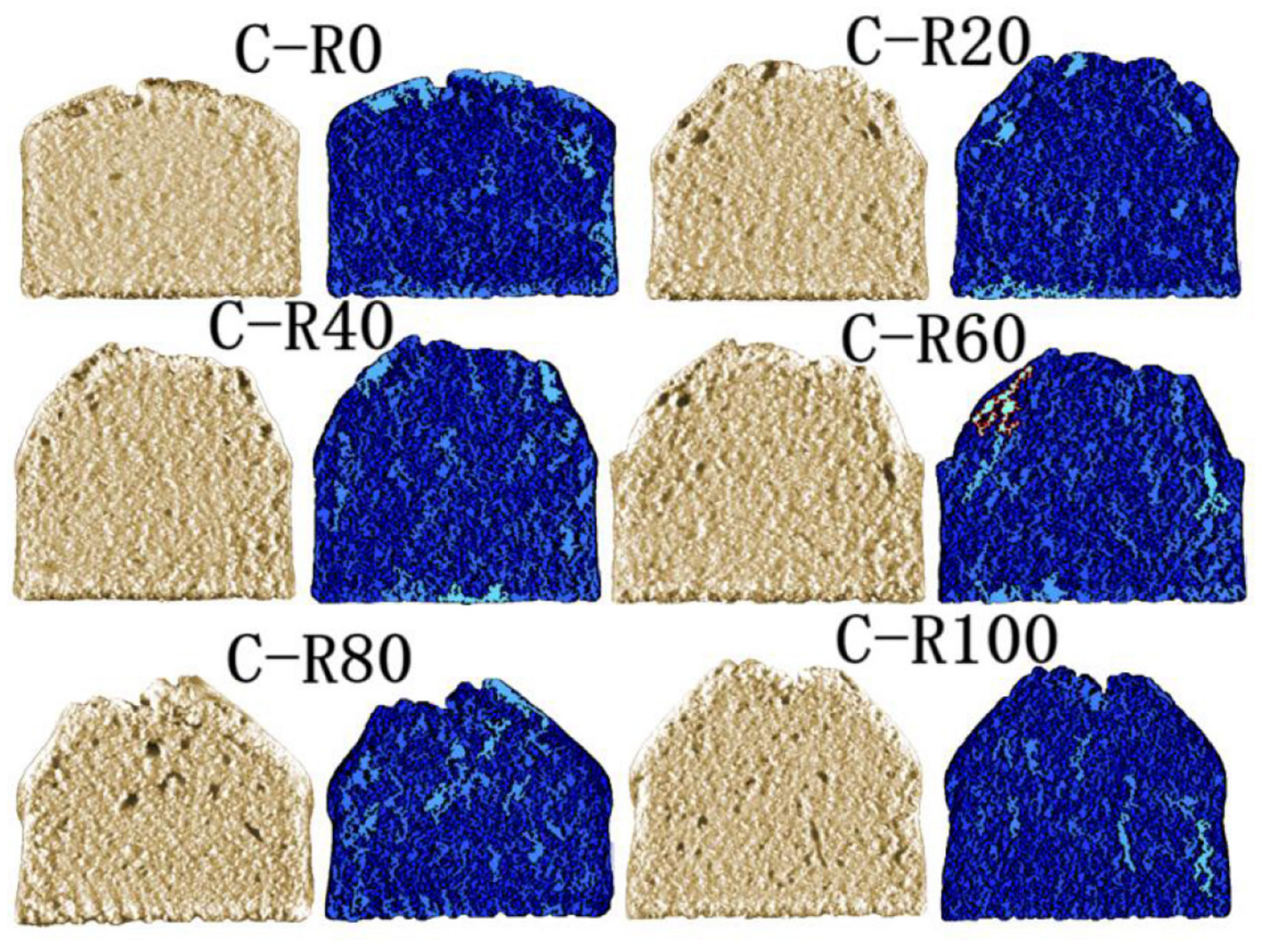
Internal structure images of cakes.

As functional fat substitutes, emulsion gels enable the reduction of saturated fat content in baked goods while modulating texture and gas retention properties (49). This study investigated the effects of IPP emulsion gel substitution for butter (RBS = 0%−100%) on cake appearance and texture through C-CELL imaging and internal structural parameter analysis (Figure 7; Table 5). Results indicate that butter substitution rates significantly regulate the microstructure and macroscopic properties of cakes. At low substitution rates (RBS = 20%−40%), the emulsion gel simulated the emulsifying function of butter via interfacial stabilization, yielding uniform pore distribution, textural softness comparable to the full-butter group, and minimal differences in surface gloss. This confirmed its feasibility in low-fat baking. Conversely, high substitution rates (RBS = 60%−100%) induced structural heterogeneities, manifested as pore collapse, significant hardness increase, and limited surface browning. These effects were attributed to the increased rigidity of the emulsion network and water-holding disparities, which disrupted the gluten-starch matrix. These findings provide a theoretical foundation for developing plant-based, low-fat cakes (50).

**Table 5:** Internal structure parameters of cakes.

**Parameter**	**C-R0**	**C-R20**	**C-R40**	**C-R60**	**C-R80**	**C-R100**
Slice area (mm^2^)	4,582.62 ± 196.54^a^	4,577.12 ± 81.73^a^	4,408.09 ± 110.36^a^	4,181.09 ± 85.94^b^	3,872.54 ± 99.56^c^	4,111.60 ± 130.13^b^
Brightness	117.77 ± 1.60^a^	92.42 ± 1.06^b^	82.90 ± 1.41^c^	72.23 ± 0.41^d^	75.79 ± 1.18^e^	73.23 ± 0.26^f^
Cell contrast	0.74 ± 0.01^a^	0.71 ± 0.01^b^	0.69 ± 0.01^b^	0.67 ± 0.01^c^	0.68 ± 0.02^c^	0.68 ± 0.01^c^
Number of cells	3,136.67 ± 133.67^a^	3,015.00 ± 351.71^ab^	2,860.00 ± 150.33^ab^	2,662.33 ± 107.62^ab^	2,831.33 ± 230.79^ab^	3,032.33 ± 121.67^ab^
Area of cells (mm^2^)	53.66 ± 0.99^a^	53.89 ± 0.53^a^	53.62 ± 0.52^a^	53.87 ± 0.67^a^	52.72 ± 0.79^b^	52.05 ± 0.53^b^
Wall thickness (mm)	0.43 ± 0.01^a^	0.44 ± 0.02^a^	0.44 ± 0.01^a^	0.44 ± 0.013^a^	0.42 ± 0.01^a^	0.42 ± 0.01^a^
Cell diameter (mm)	2.21 ± 0.14^abc^	2.43 ± 0.22^ab^	2.42 ± 0.10^ab^	2.46 ± 0.15^a^	2.13 ± 0.22^bc^	2.02 ± 0.07^c^
Cell volume (mm^3^)	7.19 ± 0.42^b^	8.17 ± 1.05^ab^	8.33 ± 0.31^ab^	8.94 ± 0.89^a^	7.45 ± 0.94^b^	7.12 ± 0.33^b^
Coarse cell volume (mm^3^)	12.75 ± 1.78^ab^	12.82 ± 1.13^ab^	13.53 ± 0.97^ab^	14.95 ± 2.02^a^	11.84 ± 2.15^b^	10.81 ± 0.88^b^

Different superscript lowercase letters in the same row indicate statistically significant differences among groups (p < 0.05).

## 4 Conclusions

IPP exhibited a broad particle size distribution (5–96 μm) and moderate wettability (three-phase contact angle, 75.62° ± 1.27°), enabling the formation of stable emulsion gels through interfacial adsorption and three-dimensional network assembly. At *w* ≥ 3%, O/W Pickering emulsion gels with *ϕ* ≥ 60% achieved a balance between mechanical strength and stability. The droplet diameter was positively correlated with ϕ and negatively correlated with w, while gel strength significantly increased with both parameters. These emulsion gels displayed solid-like viscoelastic behavior and shear-thinning properties, consistent with non-Newtonian fluid characteristics. Pickering emulsion gels prepared at *ϕ* = 60% and *w* = 4% could partially substitute butter in cake production. At RBS ≤ 60%, the cake texture, specific volume, and internal pore structure showed no significant differences compared to the full-butter control group. This study not only proposes a novel approach for utilizing immature peach (IPP) resources but also offers innovative insights for constructing food-grade Pickering emulsions.

## Data Availability

The original contributions presented in the study are included in the article/supplementary material, further inquiries can be directed to the corresponding author.
